# Effects of sampling strategy and DNA extraction on human skin microbiome investigations

**DOI:** 10.1038/s41598-019-53599-z

**Published:** 2019-11-21

**Authors:** Rie Dybboe Bjerre, Luisa Warchavchik Hugerth, Fredrik Boulund, Maike Seifert, Jeanne Duus Johansen, Lars Engstrand

**Affiliations:** 1 National Allergy Research Centre, Herlev-Gentofte Hospital, University of Copenhagen, Hellerup, Denmark; 2 Center for Translational Microbiome Research, Department of Microbiology, Tumor and Cell Biology, Karolinska Institutet, Science for Life Laboratory, Stockholm, Sweden

**Keywords:** Microbial communities, Biomarkers, Molecular medicine

## Abstract

The human skin is colonized by a wide array of microorganisms playing a role in skin disorders. Studying the skin microbiome provides unique obstacles such as low microbial biomass. The objective of this study was to establish methodology for skin microbiome analyses, focusing on sampling technique and DNA extraction. Skin swabs and scrapes were collected from 9 healthy adult subjects, and DNA extracted using 12 commercial kits. All 165 samples were sequenced using the 16S rRNA gene. Comparing the populations captured by eSwabs and scrapes, 99.3% of sequences overlapped. Using eSwabs yielded higher consistency. The success rate of library preparation applying different DNA extraction kits ranged from 39% to 100%. Some kits had higher Shannon alpha-diversity. Metagenomic shotgun analyses were performed on a subset of samples (N = 12). These data indicate that a reduction of human DNA from 90% to 57% is feasible without lowering the success of 16S rRNA library preparation and without introducing taxonomic bias. Using swabs is a reliable technique to investigate the skin microbiome. DNA extraction methodology is crucial for success of sequencing and adds a substantial amount of variation in microbiome analyses. Reduction of host DNA is recommended for interventional studies applying metagenomics.

## Introduction

The human skin is colonized by millions of bacteria, fungi and vira composing the skin microbiome. It has long been recognized that microbes are important players in skin diseases. Recently the relationship between host and skin microbes has experienced a renaissance of research activity after the rise of high-throughput DNA sequencing in 2006^1^ and subsequently increased access and continued decrease in cost. Most protocols for characterization of human microbial communities have been developed for gut microbiome studies. The skin harbors completely different niches and unique methodological challenges such as high contamination risk^2^ mainly due to low microbial biomass combined with a risk of adding contaminating microbes during handling of samples, e.g. from extraction reagents^3^. Difficulties in acquiring sufficient bacterial DNA for microbiome analysis is also an obvious challenge in skin microbiome studies^4,5^.

The most common approach to characterize the skin microbiome is amplicon sequencing of the small subunit ribosomal RNA (16S rRNA in prokaryotes) gene. This gene is ideal for community fingerprinting since it is highly conserved and possesses conserved and variable regions. Groundbreaking studies of the skin microbiome have been made with amplicon sequencing. Grice and colleagues have shown that physiologically comparable skin sites (sebaceous, moist and dry) harbor similar bacterial communities and that sampling with swabs, scrapes and punch biopsies captures the same dominant microbial components^6,7^. Optimal methodologies for conducting skin microbiome research have received increased focus (reviewed in Kong *et al*.^8^) and it is becoming apparent that multiple steps in the analyses pipeline influence the results. Concerning sampling strategy different methodologies differ not only in sampling depth and discomfort but also in biomass yield and human DNA contribution. Compared to swabs, scrapes potentially increase the biomass collected which is useful in studies with rare taxa^9^. When isolating DNA for sequencing, most scientists conducting skin microbiome research use commercial kits relying on protocols which often differ in their strategy for disrupting bacterial cells. Enzymatic treatment, thermal disruption and/or mechanical lysis are commonly used. Bead size and material are likely to influence and select certain microbial populations.

Over the past few years, whole metagenomic shotgun sequencing has become a common method for assembling genomes. This method has increased resolution and higher internal consistency^10^ compared to 16S rRNA gene sequencing and can identify bacteria to strain-level. Shotgun sequencing captures all the genetic material in a sample without a targeted amplicon step, allowing comparisons on kingdom abundances. One such study of the skin has shown that site specificity also applies to fungi, but not to eukaryotic vira^11^. The same study showed that healthy adults maintain their skin communities for up to two years. However, since the human genome is about one thousand times larger than bacterial genomes, the total DNA pool can easily become dominated by host DNA, which might obscure small microbial differences e.g. induced in interventional studies. Depleting host DNA in samples for shotgun sequencing may be a crucial approach to improve data and different approaches have recently been compared in saliva samples^12^.

While more thorough investigations of the effect of sampling procedures have been conducted for samples with high bacterial load and low amount of human DNA^13,14^, a comparable effort focusing on the skin microbiome is lacking. With a very large amount of human DNA and a particular microbiome composition, this environment presents different challenges. Here we present a comparison of two sampling strategies and 12 commercially available DNA extraction kits for investigating the skin and nasal microbiome. We amplified the 16S rRNA gene and sequenced hypervariable regions 3–4. Nasal samples were also sequenced with shotgun metagenomics to evaluate the usefulness of host DNA depletion applied in two kits by use of lysis of human cells and subsequent addition of nucleases.

## Results

### Success of 16S rRNA gene sequencing varied with DNA extraction method

Nine healthy volunteers (5 women, 4 men), aged 26 to 64 years, were included in the study. In total, 220 samples were collected, including 144 skin samples from eSwabs and scrapes (Fig. S1, Table S1), 36 nasal eSwabs, 16 *E. coli* positive controls, and 24 negative controls containing either preservation medium from eSwabs or buffer from the kits. All 220 samples were analysed with 16S rRNA gene sequencing. Quality filtering removed samples with loading mistakes or less than 5000 reads, leaving 165 samples including 137 skin and nares samples used for figures. In total, 4,017,433 reads were produced in the 137 samples. The minimal number of reads in a sample was 11,360 and the median was 27,947. Furthermore, operational taxonomic units (OTUs) present in less than 10% of the samples were removed, leaving 4814 OTUs in the 137 samples (Table S2, a complete OTU table).

We applied 12 different DNA extraction kits (Table 1) with different success rates of library preparation (Table 2). Kits number 2, 10, 11 and 12 performed poorly with rates of successful libraries ranging from 39–79%. Success in library preparation was overall independent of DNA concentrations in the samples (Table 2). However, the worst performing kit, number 11, with a success rate of 39%, also had a very low average DNA concentration of 0.09 ng/µl.

**Table 1:** Specifications of the used DNA extraction kits.

	DNA extraction kit	Kit number in this study	Storage of samples	Removal of human DNA	Thermal disruption	Chemical disruption	Mechanical disruption, bead beat	Binding to column	Washing	Elution	Storage temp.
MO BIO	BiOstic Bacteremia DNA Isolation Kit	1	−80 °C	no	70 °C	yes (no proteinase k)		yes	yes	30 µL solution CB5	−80
	Microbial DNA Isolation Kit	2	−80 °C	no	4 °C	yes (no proteinase K)		yes	yes	30 µL solution MD5	−20
	PowerLyzer UltraClean Microbial DNA Isolation Kit	3	−80 °C	no	4 °C	yes (no proteinase k)		yes	yes	30 µL solution MD5	−20
	UltraClean Tissue & Cells DNA Isolation Kit (Old LOT)	4	−20 °C	no	No	yes (high salt, proteinase K)		yes	yes	30 µL solution TD3 (no salt)	−80
	UltraClean Tissue & Cells DNA Isolation Kit (New LOT)	5	−20 °C	no	No	yes (high salt, proteinase K)		yes	yes	30 µL solution TD3 (no salt)	−80
	PowerSoil DNA isolation kit	6	−80 °C	no	4 °C	yes (no proteinase k)		yes	yes	30 µL solution C6	−80
Epicentre	MasterPure yeast DNA purification kit (+PureLink Genomic DNA kit)	7	−80 °C	no	65 °C	yes (+lysozyme, no proteinase K)		yes	yes	30 µL Milipore DNase free water	−20
QIAGEN	QIAamp DNA Investigator Kit	8	2–8 °C (max 48 hours)	no	56 °C	yes		yes	yes	30 µL buffer ATE	−20
	QIAamp DNA Microbiome Kit	9	2–8 °C (max 48 hours)	yes (benzonase)	56 °C	yes		yes	yes	50 µL buffer AVE	−20
Invitrogen	PureLink Genomic DNA Kit	10	−80 °C	no	55 °C	yes (+lysozyme)		yes	yes	35 µL elution buffer	−20
	PureLink Microbiome DNA purification	11	−80 °C	no	65 °C	yes (proteinase k)		yes	yes	30 µL S6	−20
Molzym	MolYsis™ Complete5 (Ultra-deep Microbiome prep kit)	12	−20 °C	yes (MolDNase B)	37 °C − > 56 °C − > 70 °C	yes (+2−mercaptoethanol)	no	yes	yes	40 µL deionized water (70 °C)	−20

**Table 2:** DNA concentrations after extraction, success rate of libraries prepared for 16S rRNA gene sequencing and percent of human DNA in samples from the nares.

Kit number	DNA concentration (ng/µl) total kit average	DNA concentration (ng/µl) Skin samples average	DNA concentration (ng/µl) Nares samples average	DNA concentration (ng/µl) E. Coli samples average	Successful libraries (16S)	% human DNA (shotgun)
1	1.99	0.05	12.17	3.03	100% (15/15)	90.1
2	0.67	0.02	3.76	0.34	67% (8/12)	90.3
3	0.52	0.01	0.91	6.85	94% (16/17)	85.3
4	0.51	0.00	3.30	NA	100% (13/13)	91.3
5	0.65	0.00	1.13	1.89	100% (16/16)	91.0
6	0.41	0.03	4.34	0.37	100% (12/12)	91.1
7	1.26	0.02	1.72	17.28	95% (17/18)	89.4
8	1.22	0.99	6.24	NA	95% (17/18)	89.8
9	0.09	0.01	0.06	0.68	95% (17/18)	57.4
10	1.95	0.03	8.69	8.68	78% (14/18)	91.7
11	0.09	0.02	0.27	0.60	39% (7/18)	88.8
12	0.39	0.04	0.72	2.36	79% (15/19)	89.6

Success of libraries for 16S rRNA sequencing was evaluated based on 194 samples in total, 26 samples affected by loading errors were excluded. Percent of human DNA in samples were found from 12 samples from the nares which were shotgun sequenced.

### The eSwab is a preferable method

**Figure 1:**
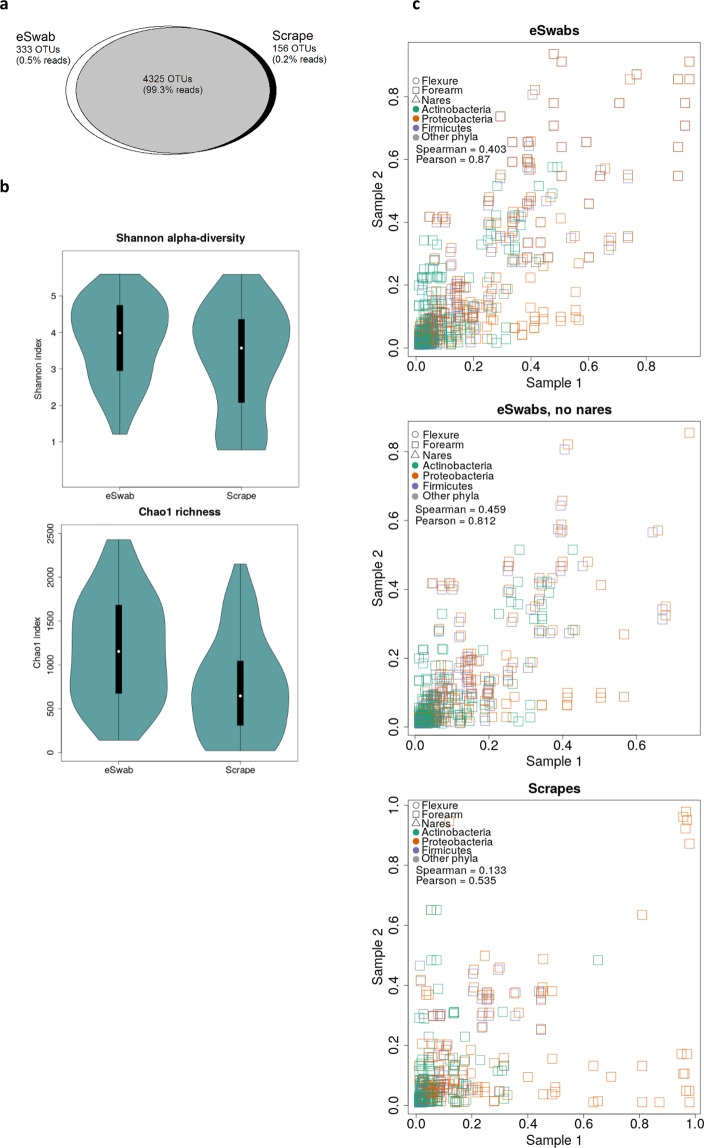
Comparison of skin sampling method. (**a**) A Venn diagram illustrating overlap of OTUs with ≥98% similarity and percent of sequence reads overlapping in parenthesis. (**b**) Violin plots illustrating Shannon alpha-diversity and Chao1 richness according to sampling method. (**c**) Scatter plots comparing the proportion of reads from a pair of samples from the same clade at the genus taxonomic level. Each sample is a pair of samples from the same skin site in the same individual, extracted with different kits. Pearson’s product moment and Spearman’s rank correlation were calculated for each plot.

We used eSwabs collecting material from the surface of the skin and scrapes collecting skin cells and microbes from the outermost part of the epidermis. There were no differences in concentrations of isolated DNA or success of library preparation between eSwabs and scrapes (data not shown). Furthermore, of the total 4814 OTUs, 4325 (89.8%) were identified with both sampling methods and these OTUs represented 3,989,311 sequence reads of the total 4,017,433 reads (99.3%) (Fig. 1a). Unique OTUs were identified using both eSwabs and scrapes. To further evaluate potential differences between microbial populations, Shannon alpha-diversity and Chao1 richness were compared (Fig. 1b). Differences between eSwabs and scrapes were found in Shannon alpha-diversity (mean scrape: 3.2; mean swab: 3.8; unadjusted p-value: 0.04), and in Chao1 richness, with lower richness in scrapes (mean scrape: 743; mean swab: 1180; unadjusted p-value: 0.00008). Notably, scrapes captured more *Pseudomonas* than eSwabs (Fig. S2). Redoing this analysis after removing all Pseudomonadales, this conclusion still holds for the richness, albeit with a higher p-value (Shannon’s p-value: 0.08; Chao1: p = 0.016). The eSwab seems to be a more consistent method than scrapes, with better Pearson’s product-moment coefficients at all taxonomic levels compared to scrapes (Figs. 1c and S3–5). Since the nare samples were only collected by eSwabs, we also made the scatter plots without those samples to rule out that inclusion of those made the scrapes seem worse off (Fig. 1c). Excluding Pseudomonadales improves Pearson’s r at all taxonomic levels, but scrapes still perform consistently worse than swabs.

### DNA extraction method captured different microbial communities

**Figure 2:**
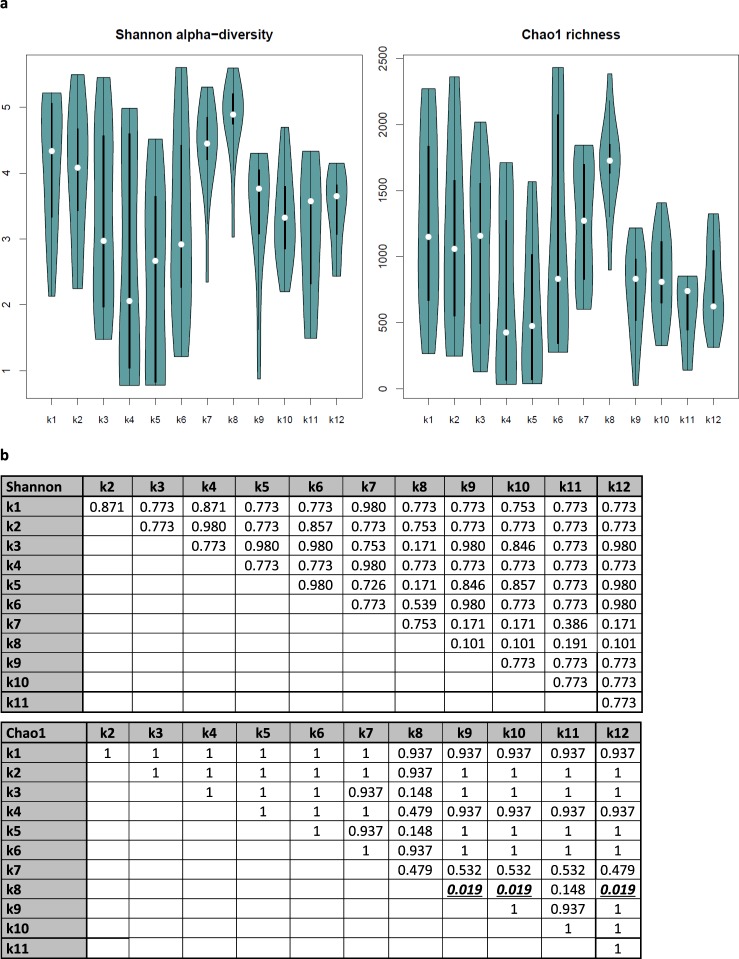
Influence of DNA extraction kit on microbiome diversity and richness. (**a**) Violin plots illustrating Shannon alpha-diversity and Chao1 richness according to DNA extraction kit. (**b**) Tables with p-values from Kruskal-Wallis-tests corrected for multiple testing by the Benjamini-Hochberg procedure, bold and underlined when statistical significance.

**Figure 3:**
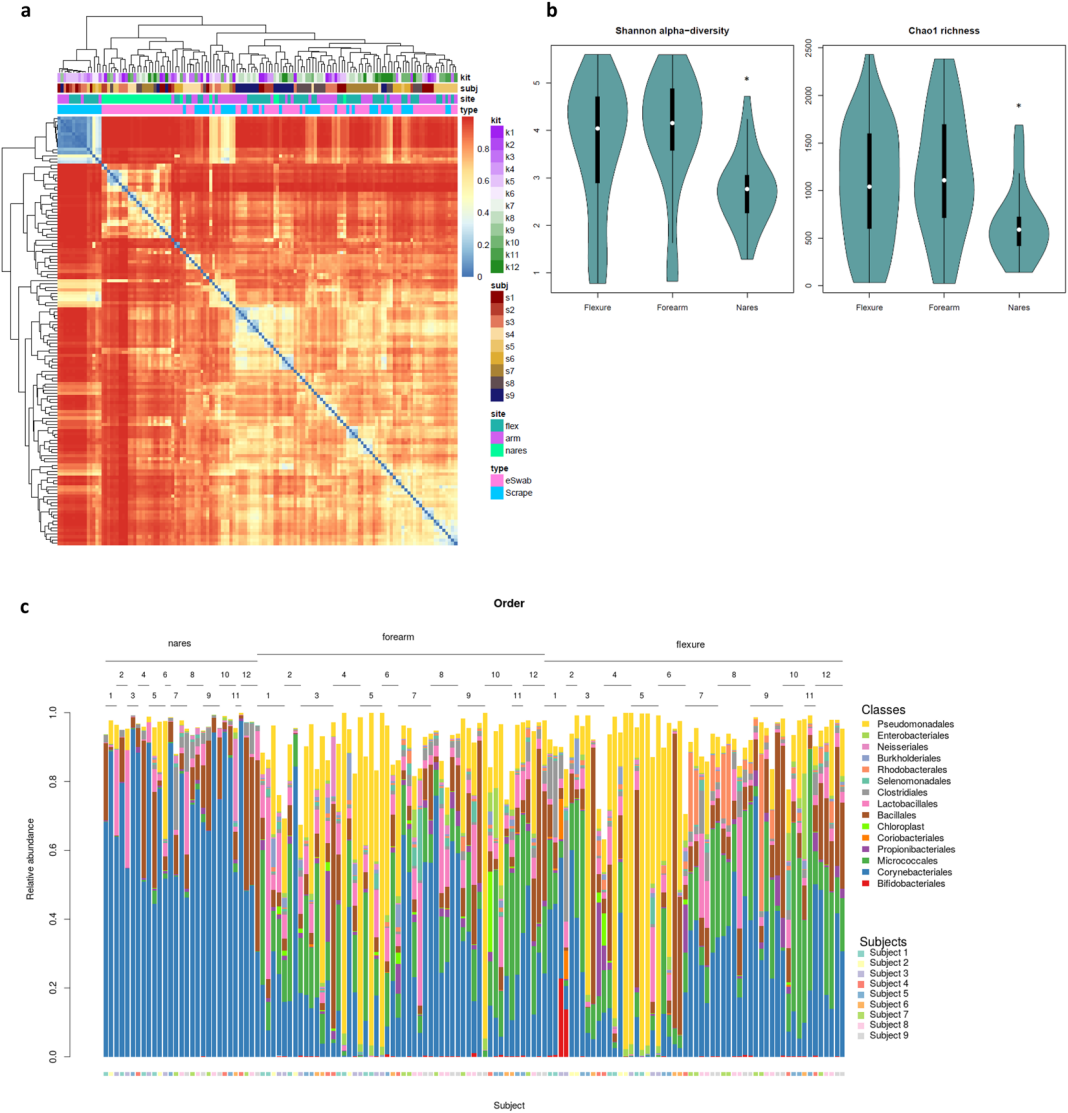
Variation by skin site. (**a**) A heatmap of Bray-Curtis distances between samples, with metadata plotted on the axis above and color code to the right. 0 indicates that samples share the same OTU and 1 that they are totally different. (**b**) Violin plots illustrating Shannon alpha-diversity and Chao1 richness according to skin site, * when statistical significance in a Kruskal-Wallis-test corrected for multiple testing by the Benjamini-Hochberg procedure (p < 0.05). (**c**) Bar charts depicting relative abundances of bacteria at the order taxonomic level. Samples are sorted by skin site and number of kit used is assigned above the charts. Individual subject numbers is indicated by the colour bar at the bottom of the figure.

The spread observed on the scatter plots (Figs. 1 and S3–5) suggests that different extraction methods capture somewhat different communities since each pair of samples on the plots comes from the same skin site in the same individual but were extracted with different kits. If the performance of all kits were the same, the points would be close to the y = x diagonal. The 12 different DNA extraction kits applied (Table 1) had some influence on Shannon alpha-diversity and Chao1 richness. Especially kit number 8 differs from most of the other kits, with high Shannon alpha-diversity and Chao1 richness (Fig. 2). Kit number 7 had this tendency as well. Notably, both kit number 7 and 8 applied a 3-mm stainless steel ball and bead beating in a Tissuelyser for mechanical disruption of bacterial cell walls and membranes (Table 1). However, kit number 10 also applied this bead beating protocol and did not yield higher Shannon alpha-diversity or Chao1 richness. Kit number 4 and 5 (same kits with different lot numbers, Table 1) had low Shannon-diversity and Chao1 richness (Fig. 2) and were dominated by Enterobacteriales (Fig. 3c), which might indicate contamination. Kits 5 and 6 also presented a large relative abundance of Pseudomonadales, also suggesting kit contamination. Our negative controls show that the main background is Pseudomonas (average 72% in all kit negative controls) and E. coli is present in most negative controls as well (average 3%) (Fig. S6). Two kits, number 8 and 10, have diverse profiles in their negative controls. Kit number 10 has a large representation (17%) of Burkholderia.

**Figure 4:**
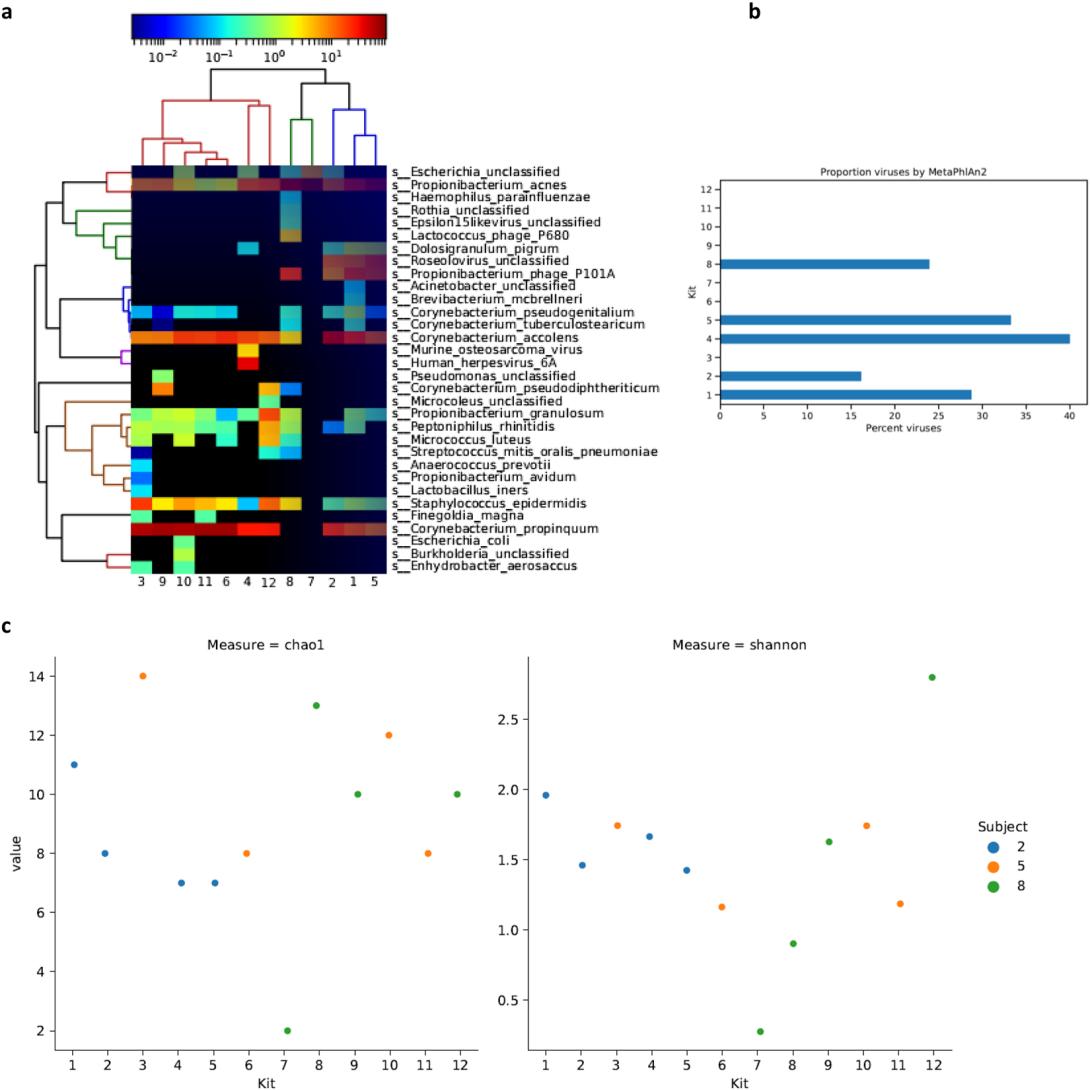
Reduction of host DNA does not influence microbial communities. (**a**) A MetaPhlAn2 clustered heatmap showing the distribution of microbes in the 12 samples, each representing one nasal sample from the kits applied (Table S2). Kit number is annotated along the x-axis and detected species-level names on the y-axis on the right side. (**b**) Percent viral DNA (x-axis) in the samples from each kit (y-axis). (**c**) Scatter plots illustrating Shannon alpha-diversity and Chao1 richness.

The heatmap (Fig. 3a) of Bray-Curtis dissimilarities did not indicate clustering according to DNA extraction kit. The subject had more influence on clustering than kit (Figs. 3a and 4).

### Variation by skin location

Evaluation of beta-diversity using Bray-Curtis Dissimilarity reveals clustering by skin site (Fig. 3a) first and then by subject. Whether samples were taken from the antecubital and popliteal fossae or volar forearm had a relatively minor impact on Shannon-alpha Diversity, Chao1 richness (Fig. 3b) and the composition of the skin microbiome (Fig. 3c). Nasal samples differed significantly from skin samples, with lower Shannon-alpha Diversity, Chao1 richness and a microbiome dominated by *Staphylococcus* (primarily *S. epidermidis*) and *Corynebacterium*. The skin also contained *Staphylococcus* and *Corynebacterium* (Fig. S2). The experimental procedures used here did not allow us to assess the impact of Cutibacterium (see Discussion for details).

### Reduction of host DNA content for shotgun sequencing

To investigate the efficacy of host DNA removal, one nasal sample from each of the 12 DNA extraction kits were also shotgun sequenced (Table S3). The samples were from subjects 2 (kit number 1, 2, 4 and 5), 5 (kit number 3, 6, 10 and 11) and 8 (kit number 7, 8, 9 and 12). A detailed description of the sequencing procedure and basic sequencing data quality measures is available in the Methods section.

Kit number 9 reduced the percent of human DNA from app. 90% to 57% (Table 2). Kit number 12 did not succeed in depleting or reducing human DNA. Importantly, using kit number 9 does not seem to introduce taxonomic biases as the sample clusters with samples from other kits (Fig. 4a) and the microbial profile is similar to samples from other kits.

The sample from kit number 7 gave the most distinct microbial profile (Fig. 4c) with a predominance of *Cutibacterium acnes* and unclassified *Escherichia* (Fig. 4a), where the latter might indicate contamination. As with 16S, the other samples were dominated by *Staphylococcus* and *Corynebacterium*. The sample from kit number 8 clusters with the one from kit number 7 and lacks a high abundance of *Corynebacterium propinquum* and *C.- accolens* (Fig. 4a).

The proportion of viral DNA (Fig. 4b) was influenced by the subject sampled. All samples from subject 2 contained viral DNA. Additionally, viral DNA was found in the sample extracted with kit number 8 from subject 8.

## Discussion

We compared sampling of skin using eSwabs and scrapes with subsequent 16S ribosomal RNA gene sequencing. A very large overlap was found, both in OTU identified and in OTU counts, indicating that these methods can be used interchangeably. This is in line with data from Chng *et al*. comparing a modified cup scrub, swab and tape-strip^10^ data from Ogai *et al*. comparing swabs and tape strips^15^ and data from Grice *et al*. comparing swabs, scrapes and punch biopsies^7^. Grice *et al*. argue that microbiota from swabs and scrapes represent a history of skin differentiation, implying that the microorganisms from deeper layers transit to the surface with differentiating skin cells. With this perspective, the outermost microbiome (live or dead) can very well indicate which processes and physiological roles the microorganism deeper in the skin^16^ may have. However, this might be too much of a simplification, as we also identify unique OTUs using each sampling method and a difference in Chao1 richness. A study applying repeated tape stripping for removal of the stratum corneum layers also show some significant differences in microbial composition between superficial and deeper layers of the stratum corneum with an increase in the relative abundance of Firmicutes (*Staphylococcus*) in the deeper layers and a decrease of Actinobacteria (*Cutibacterium*)^17^. We do not find such striking differences in microbial composition between samples of the outermost skin taken with eSwabs and scrapes going deeper in the stratum corneum. This could be due to differences in methodology, as our scrapes also capture the outermost microbiota which swabbing of specific layers after sequential tape stripping does not, but it might also be an artefact of the primers used, which do not appropriately amplify *Cutibacterium*. When sampling superficially by swabs one might overlook specific microbiota and potential interactions between microbiota and live human cells deeper in the skin. However, in our hands, data collected by eSwabs were more consistent. This technique is also less invasive and therefore more useful for certain purposes.

Many samples contained trace amounts of chloroplasts. Two of them, however, (kit 10, subject 4 and 6, flexure) contained large numbers of chloroplasts (15–25%). Since they do not consistently appear in all of kit 10’s samples, we suspect that these subjects may have considerable physical contact with plants in their everyday life or made regular use of plant-based cosmetics.

Choice of DNA extraction kit affects the observed microbial profiles, but not more than inter-individual variation. It is difficult to assess which kit comes closest to the biological truth and a limitation of this study is a lack of a proper mock community as positive control. However, kit number 4 and 5 seemed to be dominated by contaminating bacteria and are not recommended for examination of the skin microbiome.

Other factors one should consider when choosing a protocol for DNA extraction is success in sequencing and convenience of usage in a specific setting. Kits number 2, 10, 11 and 12 performed poorly with rates of successful libraries for 16S rRNA gene sequencing ranging from 39–79%. We would avoid these kits. When taking samples from patients in the clinic it is of priority that they can be stored immediately. Kit number 12 had considerable hands-on time before a storage is possible. In general, less total hands-on time is also preferable.

Kit number 9 reduced the content of human DNA from nasal samples from 90% to 57%. We were worried that adding a nuclease for reduction of host DNA would destroy free microbial DNA as well and skew the picture of microbial communities compared to extraction kits without this step. Fortunately, no taxonomic skewing was observed in 16S or shotgun data. The good correspondence between the 16S and shotgun taxonomy profile using this kit also demonstrates that reagent contamination after DNA extraction was not an issue here. Another concern was that reduction of total DNA in the samples would increase the risk of failure in library preparation, as the DNA concentrations in skin swabs generally are low, around 5 ng in total. Again, this was not a problem. Had it been a problem it would probably be advisable to sequence deeper to discover the effects of interventions rather than to reduce human DNA.

One drawback of using kit number 9 was that the total hands-on time was substantial. One recently published study compared this specific kit (Qiagen QIAamp DNA Microbiome Kit) with other methods of depleting host DNA in saliva^12^. It was found that treatment involving osmotic lysis of human cells and subsequent treatment with propidium monoazide is very efficient in removing host-derived sequences with a small taxonomic bias compared to untreated samples. Furthermore, this treatment is much cheaper than using the kit and requires fewer washing steps and less hand-on time^12^. Future studies should test this treatment on skin samples.

Whether samples were taken from popliteal and antecubital flexures or volar forearms had no impact on Shannon-alpha Diversity, Chao1 richness (Fig. 3b) or the composition of the skin microbiome (Fig. 3c). This is contradictory to the pioneer work performed by Grice *et al*.^6^ and Findley *et al*.^9^ showing that moist, dry and sebaceous areas have distinct microbial profiles. This is a general picture, and the actual differences found in these studies between the specific volar forearm and flexures are modest. Also, factors such as body composition and posture, clothing and weather can affect the moistness of the flexures. A dry flexure might be relatively similar to a volar forearm. Nasal samples differed significantly from skin samples with lower Shannon-alpha Diversity, Chao1 richness and a microbiome dominated by *Staphylococcus* (primarily *S. epidermidis*) and *Corynebacterium*. Choosing to investigate more distinct skin areas or including more areas and using metagenomics would possibly enable us to see a general pattern in differences between moist, dry and sebaceous areas.

We interpret the spread on the scatter plots as extraction methods capturing different communities. However, as we sampled non-overlapping skin areas, local differences in communities might also contribute to this spread. There are known variations in transepidermal water loss within the volar forearm, with higher values near the wrist compared to other sites of the forearm^18^. Also, recent studies show that sebum and hydration levels are predictors of microbiome composition^19^ and that the specific composition of epidermal lipids strongly affects bacterial colonization^20^. It is however not possible to circumvent this issue when comparing multiple factors, as in our study.

In addition to false negatives, DNA extraction kits can contribute with false positives, especially in environments with relatively low bacterial abundance, as the human skin. Indeed, kit 4 and 5, which had the lowest DNA extraction yield for skin samples (Table 2), also had the highest amount of Pseudomonadaceae. This family has been described to be abundant in human skin before^7^, but this observation has not been reproduced. Furthermore, Pseudomonadaceae have been found in high abundance in the “kit-ome”, i.e. the background of bacterial DNA present in DNA extraction kits and PCR reagents^2^. No thermal disruption was applied in kit number 4 and 5 (Table 1) which could result in the low amounts of isolated microbial DNA from skin samples and higher amplification of contaminating DNA. However, our main conclusions hold even when excluding all Pseudomonadales and Enterobacteriales.

After sampling and DNA extraction, another major source of bias in amplicon-based studies is primer choice. The primer pair used here was suboptimal for skin microbiome studies, since it specifically excludes Cutibacterium, as evidenced by this clade being found in shotgun, but not in amplicon samples. However, since this bias was kept constant for all samples investigated, they can still be compared. Still, future studies on the human skin microbiome will benefit from using a shotgun approach when possible (see e.g.^10^), or another primer pair for the 16S region. In this case, two approaches are possible, either selecting a different region of this gene^21,22^ or simply modifying the reverse primer to amplify the V4 region of Cutibacterium spp.^23^.

## Conclusion

Swabs and scrapes can be used interchangeably to investigate the skin microbiome. Swabs may be preferable as they are more consistent and less invasive. DNA extraction methodology is crucial for success of sequencing and adds a substantial amount of variation in microbiome analyses. However, clustering of data was more influenced by subject than kit. Using the QIAamp DNA Microbiome Kit from Qiagen, host DNA is reduced without introducing taxonomic biases, which is recommended for interventional studies applying metagenomics.

## Methods

The study was approved by the ethics committee of the Capital Region of Denmark (H-16020971). All participants signed a written informed consent form prior inclusion and any sampling. All methods were performed in accordance with relevant guidelines and regulations.

### Study participants

Nine healthy Caucasian volunteers were recruited from Hospital office staff in September 2016. Inclusion criteria were age 18 or older. Exclusion criteria were current or previous eczema, pregnancy, breastfeeding, scar tissue on sampling areas, active infections and use of antibiotics or probiotics within the past four weeks. The volunteers were instructed not to shower, use chlorinated pools, sauna, steam bath, sun tanning and topicals (e.g. moisturizers) two days before sampling at the Department of Dermatology and Allergy at Herlev and Gentofte Hospital.

### Sampling

Skin samples were collected from non-overlapping areas on the dry volar forearms and moist antecubital and popliteal fossae. The fossae were defined as the region from the flexure + /− 4 cm and the volar forearm as starting after the antecubital fossa to 4 cm from the wrist. One side of the body was randomized to sampling with eSwabs (8 samples in total) and the other with scrape (8 samples in total). Four nasal eSwabs were collected from each subject as well, giving a total of 20 samples from each subject (Fig. 1S and Table S1). For sample collection, no prior cleaning or preparation of the skin surface was done. A fresh pair of gloves were worn for each sample. The flocked swab was premoistened in either preservation medium or enzymatic lysis buffer. A timer was set at 30 sec. for rubbing the skin area. Superficial skin scrapings were obtained by taking 20 strokes in different directions at the skin with a disposable scalpel. Sample material was placed into 2 ml LoBind Eppendorf tubes containing buffer from the kit (according to manufacturer).

*E. coli* ATCC 8739–0483E7 Epower pellets (SSI Diagnostica, CFU per pellet approximately 5 × 10^7^) were suspended (according to manufacturer) in either preservation medium, buffer from the kit or enzymatic lysis buffer. Samples were either stored at −20 °C, −80 °C or processed immediately, according to DNA extraction protocol.

### DNA extraction

DNA was extracted using 12 different commercial kits (kit number 4 and 5 were similar, but had different lot numbers) according to manufactures’ protocols: 1. BiOstic Bacteremia DNA Isolation Kit (MO BIO, lot no.: BC16C25), 2. Microbial DNA Isolation Kit (MO BIO, lot no.: U16E2), 3. PowerLyzer UltraClean Microbial DNA Isolation Kit (MO BIO, lot no.: PL16C29), 4. UltraClean Tissue & Cells DNA Isolation Kit (MO BIO, lot no.: U15I14), 5. UltraClean Tissue & Cells DNA Isolation Kit (MO BIO, lot no.: U16D18), 6. PowerSoil DNA Isolation kit (MO BIO, lot no.: PS16C29), 7. MasterPure yeast DNA purification kit (Epicentre, lot no.: 0020027874), 8. QIAamp DNA Investigator Kit (QIAGEN, lot no.: 154018987), 9. QIAamp DNA Microbiome Kit (QIAGEN, lot no.: 154026306), 10. PureLink Genomic DNA Kit (Invitrogen, lot no.: 17462207), 11. PureLink Microbiome DNA purification (Invitrogen, lot. No.: 1761498), 12. MolYsis Complete5 Ultra-deep Microbiome prep kit (Molzym, lot no.: S22qKG020025). These kits were chosen because they were applied in published skin microbiome studies and/or recommended by the manufacturer to be useful for skin microbiome analysis. The combinations of kits, locations and subjects are described in Fig. S1 and Table S1.

### 16S rRNA gene sequencing

DNA concentration was determined with Quant-iT ds DNA broad range kit (Thermo Fisher Scientific, Waltham, MA, USA). The V3-V4 hypervariable region of the 16S rRNA gene was amplified using universal primers F341 and R805^24^. The PCR was performed according to the 2-step PCR protocol as described in Hugerth *et al*. 2018 with 23 µL of DNA solution as input^25^. The product was then cleaned with AMPure XP beads (Beckman Coulter, Brea, CA, USA) before being submitted to a 13 cycle barcoding reaction with Nextera XT index kit V2 (Illumina, San Diego, CA, USA) according to instructions from the manufacturer. The amplicons were sequenced on Illumina’s MiSeq platform with 2 × 300 bp reads and a cutoff value of 5000 reads was applied. A blank (negative) PCR control was amplified and sequenced with each plate.

### Operational taxonomic unit picking

After amplicon sequencing, we used Cutadapt v.1.16^26^ to remove read pairs not carrying both primers or with an average Phred score < 15. Read pairs were then merged using Vsearch v.2.6.2^27^ and excluding non-merging reads, merged pairs containing any ambiguous bases, with more than 3 expected errors over the full length or with a length <380 bp or >520 bp. We then used the unoise algorithm from Usearch v.10.0.240^28^ to denoise reads. For quantification, all merged reads were mapped back to the centroid sequences requiring at least 98% identity over the full length of the query. To differentiate between *S. aureus* and *S. epidermidis* an additional run was made with 99.8% identity clustering. Taxonomy was assigned based on the SILVA database v128^29^ using the algorithm described by Hugerth *et al*. (2018). Plant-chloroplast and mitochondrial OTUs were removed. All calculations on 16S rRNA gene data are OTU-based.

### Metagenomic sequencing and analysis

Shotgun metagenomic sequencing was performed on 12 nasal samples (Table S3, accession number table). Sequencing libraries were constructed using Rubicon ThruPLEX DNA-seq, with an average fragment size of 365 base pairs (min: 304, max: 441, stdev: 42.2). Clustering was done by ‘cBot’ and samples were sequenced on an Illumina HiSeq2500 (HiSeq Control Software 2.2.58/RTA 1.18.64) with a 2 × 126 setup using ‘HiSeq SBS Kit v4’ chemistry. The Bcl to FastQ conversion was performed using bcl2fastq_v2.19.1.403 from the CASAVA software suite. The samples were sequenced in 2 lanes with 259 and 261 million clusters each, respectively, producing an average of 40.8 million reads (min: 30.8, max: 55.2, stdev: 8.1) with on average 93.0 of bases with Q-scores greater than 30. Metagenomic sequencing data was analyzed using StaG-mwc^30,31^ version 0.2.0-dev. Reads were preprocessed with BBDuk^32^ 37.99 using the default settings defined in StaG-mwc. Host contamination was removed with BBMap^32^ v37.99 by mapping reads to a masked version of hg19 (http://seqanswers.com/forums/showthread.php?t = 42552) using the default settings defined in StaG-mwc. Taxonomic profiling was performed using MetaPhlAn2^33^ v2.7.7 using the default settings defined in StaG-mwc, with the addition of ‘-t rel_ab_w_read_stats‘ to produce estimated read counts per taxa used for downstream calculations.

### Statistical analyses

Intra-sample diversity and richness were calculated using Shannon’s entropy and Chao1 richness, respectively. Inter-sample diversity was estimated as Bray-Curtis divergence. Pairwise comparisons were performed with the Kruskal-Wallis test. All pairwise statistical comparisons were corrected for multiple testing using the Benjamini-Hochberg procedure unless otherwise stated. All calculations for 16S data were performed in R v.3.4.3, with the additional packages Vegan v.2.4-5^34^, Fossil v.0.3.7^35^ and Vioplot v.0.2 (an R package based on the original work by Hintze *et al*.^36^). Calculations and visualizations of shotgun metagenomic data was performed in a Jupyter notebook (Jupyter v4.4.0) using SciKit-bio v0.5.4, matplotlib v3.0.0^37^, seaborn v0.9.0^38^, pandas v0.22.0^39^ in Python v3.6.6.

## Supplementary information

Supplementary figures and tables

Supplementary table

## Data Availability

The sequence files and metadata for each sample in this study is publicly available at NCBI (Submission number: SUB4053477). Code used for the analysis of amplicon sequencing data is available at this repository: https://github.com/ctmrbio/Amplicon_workflows. The workflow used for the analysis of the shotgun metagenomics data is available at this repository (version 0.2.0-dev): https://github.com/boulund/stag-mwc (commit: ea3781d). Jupyter notebooks used to produce plots are available at 10.6084/m9.figshare.8319842.v1.
