# Supplementary material for: Effects of sampling strategy and DNA extraction on human skin microbiome investigations
Source: Sci Rep. 2019 Nov 21;9:17287. doi: 10.1038/s41598-019-53599-z (PMC6872721; doi:10.1038/s41598-019-53599-z)
Supplement: Supplementary file 1 — Supplementary figures and tables [file 41598_2019_53599_MOESM1_ESM.pdf]

Electronic Supplementary Material

This supplementary material has been provided by the authors to give readers additional information about their work.

Supplement to: Rie Dybboe Bjerre[1*], Luisa Warchavchik Hugerth[2], Fredrik Boulund[2], Maike Seifert[2], Jeanne Duus Johansen[1], Lars Engstrand[2]. Effects of sampling strategy and DNA extraction on human skin microbiome investigations. Scientific Reports.

*Corresponding author: rie.dybboe.bjerre@regionh.dk

Affiliations:

[1]National Allergy Research Centre, Herlev-Gentofte Hospital, University of Copenhagen, Denmark
[2]Department of Microbiology, Tumor and Cell Biology, Karolinska Institutet, Clinical Genomics Facility, Science for Life Laboratory, Stockholm, Sweden

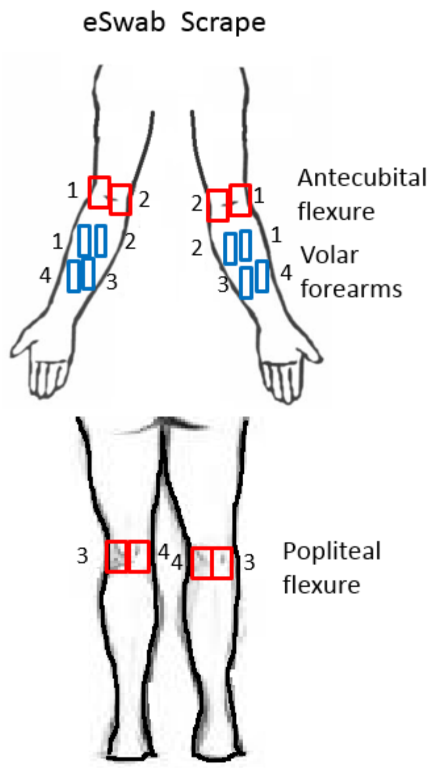

Figure S1. Skin sampling. Skin samples were collected from non-overlapping areas (illustrated with boxes) on the dry volar forearms and the moist antecubital and popliteal fossae. Each fossa was divided in two and volar forearm in four as illustrated. Kit number is exemplified with the numbers next to the boxes.
One side of the body was randomized to sampling with eSwabs (8 samples in total) and the other with scrape (8 samples in total). Four nasal eSwabs were collected from each subject as well, giving a total of 20 samples from each subject.

Sample	eSwab or scrape	Kit no.											
		5	4	1	2	3	6	11	10	12	9	8	7
Neg. Ctrl.	eSwab	1	4	7	10	73	76	79	82	145	148	151	154
Neg. Ctrl.	Scrape	2	5	8	11	74	77	80	83	146	149	152	155
Extraction Ctrl., E. coli		3e	6e	9	12	75	78	81	84	147e	150e	153	156
		3s	6s							147s	150s		
Nare	eSwab	17	22	27	32	89	94	99	104	161	166	171	176
		37	42	47	52	109	114	119	124	181	186	191	196
		57	62	67	72	129	134	139	144	201	206	211	216
Flexure, moist	eSwab	13	18	23	28	85	90	95	100	157	162	167	172
		33	38	43	48	105	110	115	120	177	182	187	192
		53	58	63	68	125	130	135	140	197	202	207	212
	Scrape	15	20	25	30	87	92	97	102	159	164	169	174
		35	40	45	50	107	112	117	122	179	184	189	194
		55	60	65	70	127	132	137	142	199	204	209	214
Volar forearm, dry	eSwab	14	19	24	29	86	91	96	101	158	163	168	173
		34	39	44	49	106	111	116	121	178	183	188	193
		54	59	64	69	126	131	136	141	198	203	208	213
	Scrape	16	21	26	31	88	93	98	103	160	165	170	175
		36	41	46	51	108	113	118	123	180	185	190	195
		56	61	66	71	128	133	138	143	200	205	210	215

Subject 1
Subject 2
Subject 3
Subject 4
Subject 5
Subject 6
Subject 7
Subject 8
Subject 9

Table S1: Metadata table. Each extraction kit was tested on samples from three subjects (colour). The letter "e" after some samples refers to suspension of E. Coli in preservation buffer from the eSwab and the letter "s" refers to suspension of E. Coli in kit buffer.

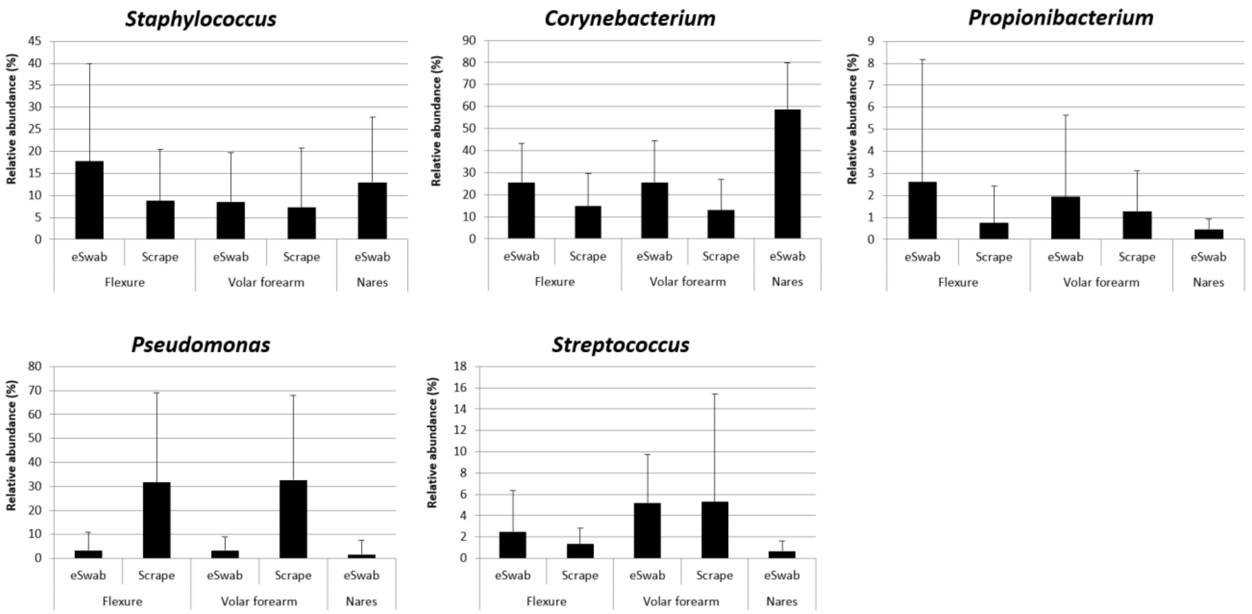

Figure S2. Relative abundances of common bacterial residents in skin. Bar charts with standard deviations depicting relative abundances of selected bacterial genera. Divided according to skin site (flexure, volar forearm, nares) and sampling method (eSwab, scrape). Pseudomonas is most likely contamination.

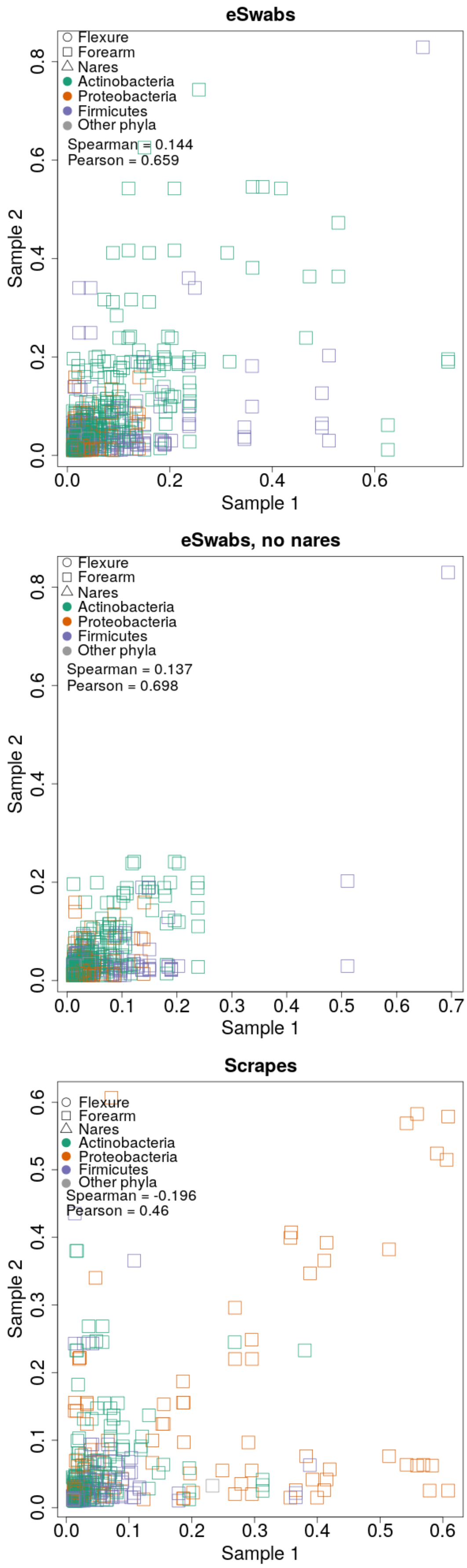

Figure S3: Community dissimilarities at OTU level. Scatter plots comparing the proportion of reads from a pair of samples from the same clade at the OTU taxonomic level. Each sample is a pair of samples from the same skin site in the same individual, extracted with different kits. Pearson's product moment and Spearman's rank correlation were calculated for each plot.

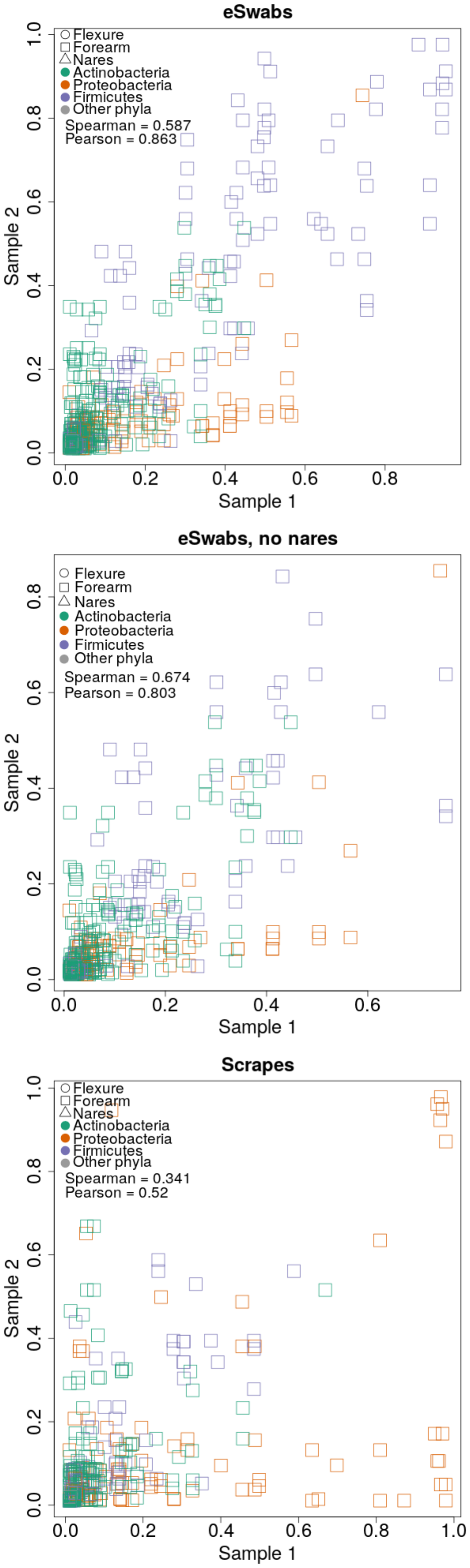

Figure S4: Community dissimilarities at family level. Scatter plots comparing the proportion of reads from a pair of samples from the same clade at the family taxonomic level. Each sample is a pair of samples from the same skin site in the same individual, extracted with different kits. Pearson's product moment and Spearman's rank correlation were calculated for each plot.

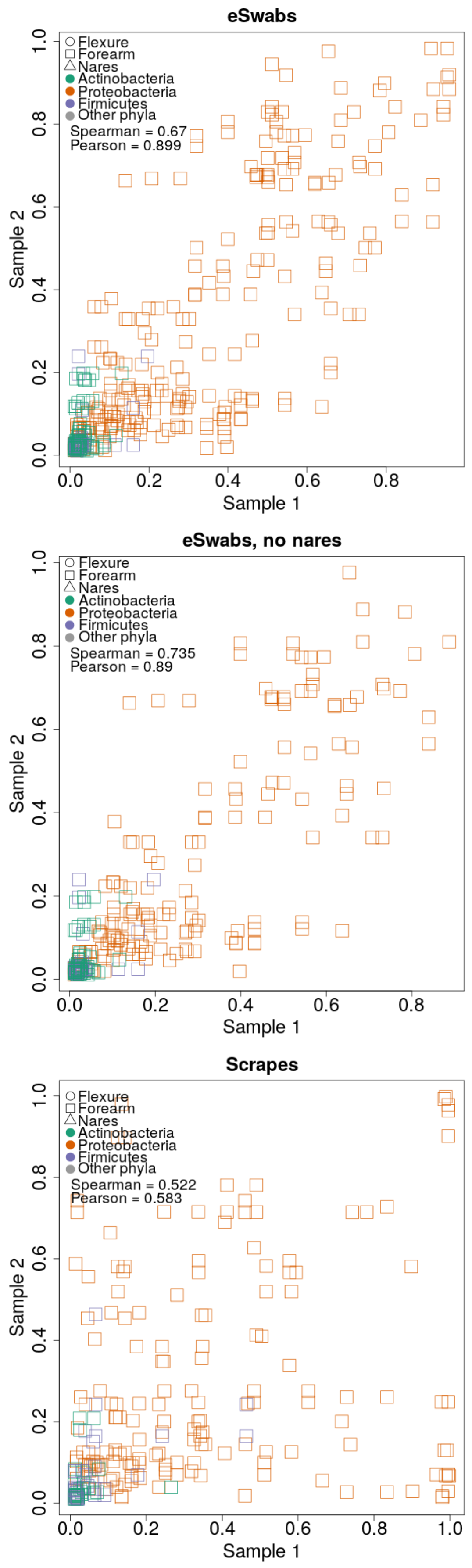

Figure S5: Community dissimilarities at class level. Scatter plots comparing the proportion of reads from a pair of samples from the same clade at the class taxonomic level. Each sample is a pair of samples from the same skin site in the same individual, extracted with different kits. Pearson's product moment and Spearman's rank correlation were calculated for each plot.

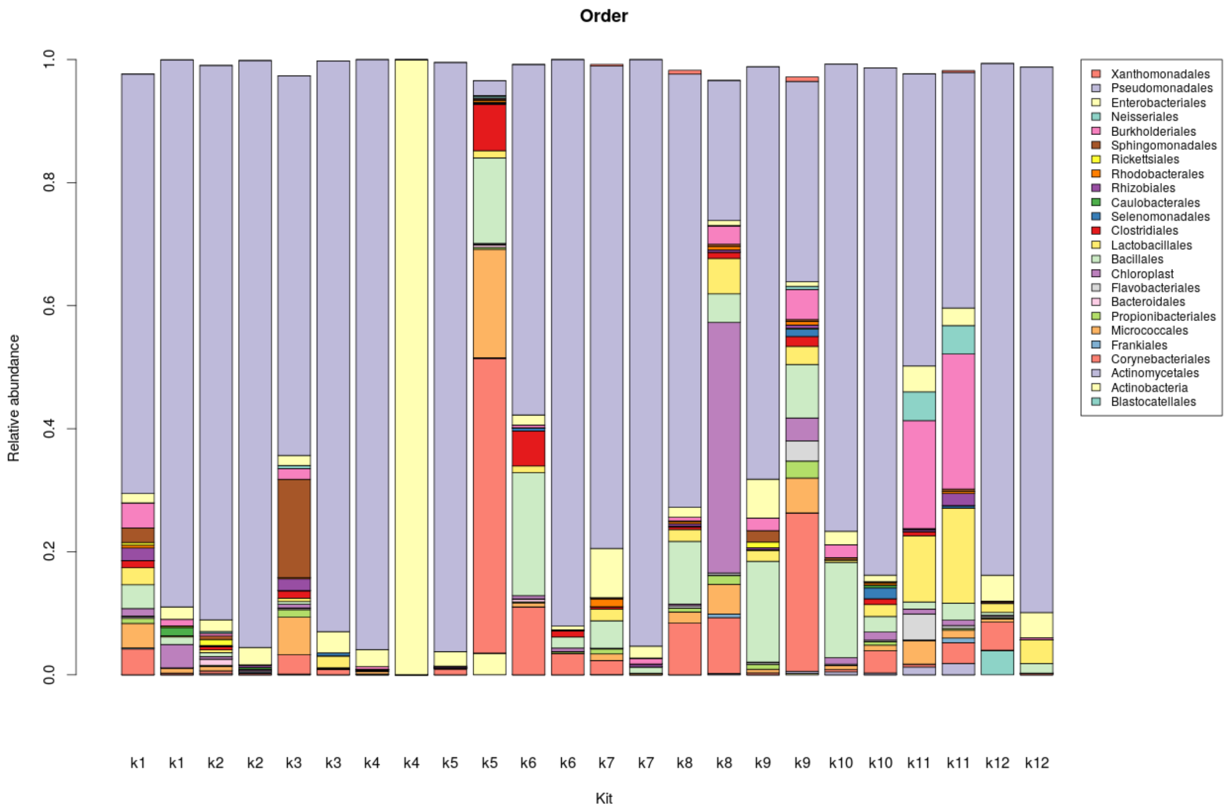

Figure S6: Relative abundances in negative controls. Bar charts depicting relative abundances of bacteria at the order taxonomic level in our negative control samples from each kit.

Accession	Sample name
SRR7294131	Neg ctrl eSwab k5
SRR7294130	Neg ctrl Scrape k5
SRR7294133	Ecoli ctrl eSwab k5
SRR7294132	Ecoli ctrl Scrape k5
SRR7294127	Neg ctrl eSwab k4
SRR7294126	Neg ctrl eSwab k1
SRR7294129	Neg ctrl Scrape k1
SRR7294128	Ecoli ctrl k1
SRR7294135	Ecoli ctrl k2
SRR7294134	eSwab Antecubital Flexure moist s1 k5
SRR7294163	eSwab Volar Forearm dry s1 k5
SRR7294162	Scrape Antecubital Flexure moist s1 k5
SRR7294161	Scrape Volar Forearm dry s1 k5
SRR7294160	eSwab Nare s1 k5
SRR7294159	eSwab Antecubital Flexure moist s1 k4
SRR7294158	eSwab Volar Forearm dry s1 k4
SRR7294157	Scrape Antecubital Flexure moist s1 k4
SRR7294156	Scrape Volar Forearm dry s1 k4
SRR7294165	eSwab Nare s1 k4
SRR7294164	eSwab Popliteal Flexure moist s1 k1
SRR7294038	eSwab Volar Forearm dry s1 k1
SRR7294039	Scrape Popliteal Flexure moist s1 k1
SRR7294036	Scrape Volar Forearm dry s1 k1
SRR7294037	eSwab Nare s1 k1
SRR7294034	eSwab Volar Forearm dry s1 k2
SRR7294035	eSwab Nare s1 k2
SRR7294032	Scrape Antecubital Flexure moist s2 k5
SRR7294033	eSwab Nare s2 k5
SRR7294030	eSwab Antecubital Flexure moist s2 k4
SRR7294031	eSwab Volar Forearm dry s2 k4
SRR7294196	eSwab Antecubital Flexure moist s2 k4
SRR7294195	Scrape Volar Forearm dry s2 k1
SRR7294198	eSwab Nare s2 k1
SRR7294197	eSwab Popliteal Flexure moist s2 k2
SRR7294192	eSwab Volar Forearm dry s2 k2
SRR7294191	Scrape Popliteal Flexure moist s2 k2
SRR7294194	eSwab Antecubital Flexure moist s3 k5
SRR7294193	eSwab Volar Forearm dry s3 k5
SRR7294190	Scrape Antecubital Flexure moist s3 k5
SRR7294189	Scrape Volar Forearm dry s3 k5
SRR7294102	eSwab Nare s3 k5
SRR7294103	eSwab Volar Forearm dry s3 k4
SRR7294104	Scrape Antecubital Flexure moist s3 k4

SRR7294105	Scrape Volar Forearm dry s3 k4
SRR7294098	eSwab Nare s3 k4
SRR7294099	eSwab Popliteal Flexure moist s3 k1
SRR7294100	eSwab Volar Forearm dry s3 k1
SRR7294101	Scrape Popliteal Flexure moist s3 k1
SRR7294106	Scrape Volar Forearm dry s3 k1
SRR7294107	eSwab Nare s3 k1
SRR7294068	eSwab Volar Forearm dry s3 k2
SRR7294067	Scrape Popliteal Flexure moist s3 k2
SRR7294066	Scrape Volar Forearm dry s3 k2
SRR7294065	eSwab Nare s3 k2
SRR7294072	Neg ctrl eSwab k3
SRR7294071	Neg ctrl Scrape k3
SRR7294070	Ecoli ctrl k3
SRR7294069	Neg ctrl eSwab k6
SRR7294063	Neg ctrl Scrape k6
SRR7294062	Ecoli ctrl k6
SRR7294138	Neg ctrl eSwab k11
SRR7294139	Neg ctrl Scrape k11
SRR7294136	Ecoli ctrl k11
SRR7294137	Neg ctrl eSwab k10
SRR7294142	Neg ctrl Scrape k10
SRR7294143	Ecoli ctrl k10
SRR7294140	eSwab Antecubital Flexure moist s4 k3
SRR7294141	eSwab Volar Forearm dry s4 k3
SRR7294144	Scrape Antecubital Flexure moist s4 k3
SRR7294145	Scrape Volar Forearm dry s4 k3
SRR7294109	eSwab Nare s4 k3
SRR7294108	eSwab Antecubital Flexure moist s4 k6
SRR7294111	Scrape Volar Forearm dry s4 k6
SRR7294110	eSwab Popliteal Flexure moist s4 k11
SRR7294113	eSwab Volar Forearm dry s4 k11
SRR7294112	Scrape Popliteal Flexure moist s4 k11
SRR7294115	Scrape Volar Forearm dry s4 k11
SRR7294114	eSwab Nare s4 k11
SRR7294117	eSwab Popliteal Flexure moist s4 k10
SRR7294116	eSwab Volar Forearm dry s4 k10
SRR7294064	Scrape Popliteal Flexure moist s4 k10
SRR7294097	Scrape Volar Forearm dry s4 k10
SRR7294073	eSwab Nare s4 k10
SRR7294074	eSwab Antecubital Flexure moist s5 k3
SRR7294075	eSwab Volar Forearm dry s5 k3
SRR7294121	Scrape Antecubital Flexure moist s5 k3
SRR7294205	Scrape Volar Forearm dry s5 k3
SRR7294086	eSwab Nare s5 k3

SRR7294040	eSwab Antecubital Flexure moist s5 k6
SRR7294041	Scrape Antecubital Flexure moist s5 k6
SRR7294125	Scrape Volar Forearm dry s5 k6
SRR7294124	eSwab Nare s5 k6
SRR7294123	eSwab Volar Forearm dry s5 k11
SRR7294122	Scrape Popliteal Flexure moist s5 k11
SRR7294120	Scrape Volar Forearm dry s5 k11
SRR7294119	eSwab Popliteal Flexure moist s5 k10
SRR7294118	eSwab Volar Forearm dry s5 k10
SRR7294180	Scrape Popliteal Flexure moist s5 k10
SRR7294169	Scrape Volar Forearm dry s5 k10
SRR7294168	eSwab Nare s5 k10
SRR7294154	eSwab Antecubital Flexure moist s6 k3
SRR7294155	eSwab Volar Forearm dry s6 k3
SRR7294152	Scrape Volar Forearm dry s6 k3
SRR7294153	eSwab Antecubital Flexure moist s6 k6
SRR7294150	eSwab Volar Forearm dry s6 k6
SRR7294151	Scrape Antecubital Flexure moist s6 k6
SRR7294148	eSwab Volar Forearm dry s6 k10
SRR7294149	Scrape Popliteal Flexure moist s6 k10
SRR7294146	Scrape Volar Forearm dry s6 k10
SRR7294147	eSwab Nare s6 k10
SRR7294175	Neg ctrl eSwab k12
SRR7294174	Neg ctrl Scrape k12
SRR7294177	Ecoli ctrl Scrape k12
SRR7294176	Neg ctrl eSwab k9
SRR7294171	Neg ctrl Scrape k9
SRR7294170	Ecoli ctrl eSwab k9
SRR7294173	Ecoli ctrl Scrape k9
SRR7294172	Neg ctrl eSwab k8
SRR7294167	Neg ctrl Scrape k8
SRR7294166	Ecoli ctrl k8
SRR7294056	Neg ctrl eSwab k7
SRR7294057	Neg ctrl Scrape k7
SRR7294058	eSwab Volar Forearm dry s7 k12
SRR7294059	Scrape Antecubital Flexure moist s7 k12
SRR7294052	eSwab Nare s7 k12
SRR7294053	eSwab Antecubital Flexure moist s7 k9
SRR7294054	Scrape Antecubital Flexure moist s7 k9
SRR7294055	Scrape Volar Forearm dry s7 k9
SRR7294060	eSwab Nare s7 k9
SRR7294061	eSwab Popliteal Flexure moist s7 k8
SRR7294090	eSwab Volar Forearm dry s7 k8
SRR7294089	Scrape Popliteal Flexure moist s7 k8
SRR7294088	eSwab Nare s7 k8

SRR7294087	eSwab Popliteal Flexure moist s7 k7
SRR7294094	eSwab Volar Forearm dry s7 k7
SRR7294093	Scrape Popliteal Flexure moist s7 k7
SRR7294092	Scrape Volar Forearm dry s7 k7
SRR7294091	eSwab Nare s7 k7
SRR7294096	eSwab Antecubital Flexure moist s8 k12
SRR7294095	Scrape Antecubital Flexure moist s8 k12
SRR7294044	Scrape Volar Forearm dry s8 k12
SRR7294045	eSwab Nare s8 k12
SRR7294042	eSwab Antecubital Flexure moist s8 k9
SRR7294043	eSwab Volar Forearm dry s8 k9
SRR7294048	Scrape Antecubital Flexure moist s8 k9
SRR7294049	Scrape Volar Forearm dry s8 k9
SRR7294046	eSwab Nare s8 k9
SRR7294047	eSwab Popliteal Flexure moist s8 k8
SRR7294050	eSwab Volar Forearm dry s8 k8
SRR7294051	Scrape Popliteal Flexure moist s8 k8
SRR7294077	Scrape Volar Forearm dry s8 k8
SRR7294076	eSwab Nare s8 k8
SRR7294079	eSwab Popliteal Flexure moist s8 k7
SRR7294078	eSwab Volar Forearm dry s8 k7
SRR7294081	Scrape Popliteal Flexure moist s8 k7
SRR7294080	Scrape Volar Forearm dry s8 k7
SRR7294083	eSwab Nare s8 k7
SRR7294082	eSwab Antecubital Flexure moist s9 k12
SRR7294085	eSwab Volar Forearm dry s9 k12
SRR7294084	Scrape Antecubital Flexure moist s9 k12
SRR7294181	Scrape Volar Forearm dry s9 k12
SRR7294182	eSwab Nare s9 k12
SRR7294183	eSwab Antecubital Flexure moist s9 k9
SRR7294184	eSwab Volar Forearm dry s9 k9
SRR7294185	Scrape Antecubital Flexure moist s9 k9
SRR7294186	Scrape Volar Forearm dry s9 k9
SRR7294187	eSwab Popliteal Flexure moist s9 k8
SRR7294188	eSwab Volar Forearm dry s9 k8
SRR7294178	Scrape Popliteal Flexure moist s9 k8
SRR7294179	Scrape Volar Forearm dry s9 k8
SRR7294204	eSwab Nare s9 k8
SRR7294203	eSwab Popliteal Flexure moist s9 k7
SRR7294202	eSwab Volar Forearm dry s9 k7
SRR7294201	Scrape Popliteal Flexure moist s9 k7
SRR7294200	Scrape Volar Forearm dry s9 k7
SRR7294199	eSwab Nare s9 k7
SRR9696275	Shotgun metagenomics of eSwab Nare s2 k5
SRR9696276	Shotgun metagenomics of eSwab Nare s2 k4

SRR9696277	Shotgun metagenomics of eSwab Nare s5 k3
SRR9696278	Shotgun metagenomics of eSwab Nare s5 k6
SRR9696284	Shotgun metagenomics of eSwab Nare s8 k12
SRR9696283	Shotgun metagenomics of eSwab Nare s8 k9
SRR9696281	Shotgun metagenomics of eSwab Nare s2 k1
SRR9696282	Shotgun metagenomics of eSwab Nare s2 k2
SRR9696273	Shotgun metagenomics of eSwab Nare s5 k11
SRR9696274	Shotgun metagenomics of eSwab Nare s5 k10
SRR9696279	Shotgun metagenomics of eSwab Nare s8 k8
SRR9696280	Shotgun metagenomics of eSwab Nare s8 k7

Table S3: Accession number table. Sample name and SRA identifier.
